# The Impairment of Wound Healing Process is Correlated With Abnormalities of TNF-α Production by Peritoneal Exudate Cells in Obstructive Jaundiced Rats

**DOI:** 10.1155/2000/82905

**Published:** 2000-01

**Authors:** Janusz Dawiskiba, Danuta Kwiatkowska, Michał Zimecki, Pawel Kornafel, Wanda Tyran, Elżbieta Czapiiska, Zdzisław Woźniak

**Affiliations:** 1 First Clinic of Surgery Wrocław Poland; 2 Department of Biochemistry Medical University of Wrocław Wrocław Poland; 3 Institute of Immunology and Experimental Therapy of Polish Academy of Sciences Wrocław Poland; 4 Department of Pathology Medical University of Wrocław Wrocław Poland; 5 First Clinic of Surgery Medical University in Wrocław Poniatowski Str. 2 Wrocław 50-326 Poland

## Abstract

The wound healing process and production of tumour necrosis factor alpha (TNF-α) by peritoneal cells of 7-day and 14-day obstructive jaundice (OJ) and sham-operated rats were investigated. In the study the skin wound breaking strength was measured, In addition such histological and biochemical parameters as fibroblast and endothelial cell proliferation, inflammatory cell infiltration and hydroxyproline content were evaluated in polyurethane sponge discs implanted subcutaneously into rats. TNF-α production by peritoneal exudate cells (PEC), both spontaneous and lipopolysaccharide (LPS)- induced was determined by a bioassay. In OJ rats the process of both early as well as late phase of healing was impaired. The breaking strength of skin wound was decreased, the fibroblast and endothelial cell proliferation and collagen deposition, as well as hydroxyproline content were diminished. In 7 day OJ the numbers of inflammatory cells in the implants were lowered with a subsequent slight increase on day 14 of OJ. The spontaneous and LPS induced TNF- α production by PEC were significantly higher in 7 day OJ as compared with sham-operated controls. On day 14 of OJ the LPS-induced TNF-α level was, in contrast, much lower and did not differ much from the spontaneous TNF-α production. We conclude that the impairment of wound healing in OJ results from disturbances in functioning of the immune system caused by systemic endotoxaemia.


*HPB Surgery*, 2000, Vol. 11, pp. 311–318
Reprints available directly from the publisher
Photocopying permitted by license only

© 2000 OPA (Overseas Publishers Association) N.V.
Published by license under
the Harwood Academic Publishers imprint,
part of The Gordon and Breach Publishing Group.
Printed in Malaysia.

### The Impairment of Wound Healing Process is Correlated with Abnormalities of TNF-$\alpha$ Production by Peritoneal Exudate Cells in Obstructive Jaundiced Rats

JANUSZ DAWISKIBA[a,*], DANUTA KWIATKOWSKA[b], MICHAŁ ZIMECKI[c], PAWEŁ KORNAFEL[a], WANDA TYRAN[b], ELŻBIETA CZAPIŃSKA[b] and ZDZISŁAW WOŹNIAK[d]

[a] *First Clinic of Surgery,* [b] *Department of Biochemistry, Medical University of Wrocław,* [c] *Institute of Immunology and Experimental Therapy of Polish Academy of Sciences,* [d] *Department of Pathology, Medical University of Wrocław, Wrocław, Poland*

*(Received 18 December 1998; In final form 10 April 1999)*

**The wound healing process and production of tumour necrosis factor alpha (TNF-α) by peritoneal cells of 7-day and 14-day obstructive jaundice (OJ) and sham-operated rats were investigated. In the study the skin wound breaking strength was measured. In addition such histological and biochemical parameters as fibroblast and endothelial cell proliferation, inflammatory cell infiltration and hydroxyproline content were evaluated in polyurethane sponge discs implanted subcutaneously into rats. TNF-α production by peritoneal exudate cells (PEC), both spontaneous and lipopolysaccharide (LPS)–induced was determined by a bioassay. In OJ rats the process of both early as well as late phase of healing was impaired. The breaking strength of skin wound was decreased, the fibroblast and endothelial cell proliferation and collagen deposition, as well as hydroxyproline content were diminished. In 7 day OJ the numbers of inflammatory cells in the implants were lowered with a subsequent slight increase on day 14 of OJ. The spontaneous and LPS induced TNF-α production by PEC were significantly higher in 7 day OJ as compared with sham-operated controls. On day 14 of OJ the LPS-induced TNF-α level was, in contrast, much lower and did not differ much from the spontaneous TNF-α production. We conclude that the impairment of wound healing in OJ results from disturbances in functioning of the immune system caused by systemic endotoxaemia.**

*Keywords:* Bile duct obstruction, wound healing, breaking strength, hydroxyproline, TNF-alpha

### INTRODUCTION

Many clinical observations have shown frequent occurrence of postoperative septic and organ complications in patients with obstructive jaundice [1,2]. Experimental studies on animal models confirmed those findings [3,4]. Postoperative mortality in OJ patients is estimated as 5–25% of cases. Sepsis, bleeding disorders and renal failure are the main causes of death [5]. Many clinical reports have demonstrated impairment of the wound healing process in

*Address for correspondence. First Clinic of Surgery Medical University in Wrocław, 50-326 Wrocław, Poniatowski Str. 2, Poland.

patients with extrahepatic cholestasis resulting in incidence of abdominal wound dehiscence or incisional abdominal hernia formation [6,7]. General disturbances of the defence mechanism, particularly of the cellular immunity, may be a principal cause of these complications [8,9]. The mechanisms causing these complications have been, however, not fully understood until now. The primary cause of these disorders seems to be systemic endotoxaemia as a consequence of reticulo-endothelial system (RES) function impairment. The liver and spleen are particularly affected [7,10,11]. Numerous experimental studies confirmed a key role for bile acids in prevention of bacteria translocation into mesenteric lymph nodes and development of endotoxaemia [12,13]. Endotoxin is a potent stimulant and even at subclinical concentrations, activates the immune system, including monocytes, tissue macrophages and granulocytes [8,14]. Induction of the acute phase response triggers activation and release of proinflammatory cytokines, mainly TNF-$\alpha$, interleukin-1 and interleukin-6, which seem to play a principal role in the wound healing process [15]. However, their roles remain still poorly defined.

Healing of the wound is a dynamic and complex process and the clinical evaluation of its early and late phase may include biochemical [6,16], immunological [15], histopathological [15] and mechanical [16,17] studies. Some authors suggest that defective wound healing in OJ patients relates to general metabolic disturbances caused by malnutrition [18], but recent studies did not confirm those presumptions [8,17]. The activity of prolylhydroxylase at the marginal side of the wound, the enzyme being an indicator of collagen synthesis, was deeply depressed in patients with extrahepatic cholestasis. Enzyme activity returned to normal value after biliary drainage [6].

Beside changes in the intensity of some biochemical parameters during the course of OJ, the immunological disturbances seem to be of a particular importance. As a result of endotoxaemia, macrophages acquire tolerance to endotoxin and produce less TNF-alpha [19,20].

The wound healing process is still not entirely elucidated and the results of the effect of extrahepatic cholestasis on the healing process have been equivocal, sometimes conflicting and controversial. Therefore, we decided to evaluate the effect of extrahepatic cholestasis on the wound healing on days 7 and 14 following elicitation of OJ. In addition, the ability of peritoneal cells with regard to spontaneous and LPS-induced production was determined.

### MATERIALS AND METHODS

Male, inbred rats of the Buffalo strain, weighing 200–240 g, were maintained in stable conditions with free access to water and commercial food.

All surgical procedures were performed under light ether anaesthesia in clean, but not sterile conditions as described elsewhere [3]. In the first part of the experiment the mechanical study of breaking strength of the surgical skin wound in 1-week and in 2-week and in sham-operated controls was carried out. The breaking strength of wounds was measured by tension tests with the use of endurance machine FPZ-100/1 with atest, (No prod. 28/85, Germany). In the second part of the study a histopathological and biochemical study of wound healing in 1-week and in 2-week OJ and in sham-operated controls was performed. We used a polyurethane sponge (Codogard R.–Tricomed, Łódź, Poland, art. No SWW 2025-239-000-50-8) sterilized in oxide ethylene. The polyurethane sponge disks, 10 mm of diameter, were implanted by two longitudinal skin incisions of 1 cm length in the dorsal region. After excision, on day 7 and 14, one implant was fixed in 5% buffered formaldehyde, a paraffin embedded sample was made and stained with hematoxyline-eosine (H-E) and Van Gieson. Microscopic estimation using a semiquantitative 3-grade scale (slight, moderate and significant) evaluated the number of inflammatory cells, proliferation of fibroblasts and endothelial cells and collagen deposition. The second polyurethane sponge was immediately frozen at −20°C, and hydroxyproline content was estimated later according to Woessner [21]. At the end of the experiment the abdominal and thoracic cavities were opened, the liver and the CBD macroscopically evaluated and blood for bilirubin estimation was taken by a direct cardiac puncture.

### Isolation of Serum and Peritoneal Cells

Peritoneal cells were obtained by lavage of the peritoneal cavity with cold Hanks medium containing heparin. For cell culture PEC were resuspended at a concentration of $10^6$/ml of culture medium consisting of RPMI 1640, glutamine, sodium pyruvate, 2-mercaptoethanol, antibiotics and 10% fetal calf serum (Gibco). Spleen cells were prepared in the same medium at a concentration of $10^7$/ml.

### Induction of Cytokines in Cell Cultures

PEC were placed in 1 ml aliquots in 24-well culture plates ($10^6$/ml) in the culture medium. Half of the cultures were untreated for measurement of spontaneous cytokine production, the other half was treated with 5 ug/ml of LPS from E.coli (Sigma, St. Louis, USA). After 24 hours of incubation (5% $CO_2$, 37°C) the supernatants were harvested and frozen at −80°C until cytokine determination.

Determination of TNF-alpha activity was performed according to Espevik *et al.* [22]. Cell proliferation was evaluated using MTT colorimetric method [23].

### Statistics

The results of the studied groups were expressed as a mean values with standard deviation or median for central and asymetrical value distributions. The differences were determined using Students'-t tests or D max. test at the significance level of 0,05.

In the statistical evaluation of the results with regard to TNF-$\alpha$ one way analysis of variance for completely randomized block design for 3 levels (influence of the studied subgroups of rats, presence of OJ and its persistance) was applied. In other studies t-Student's test, Cox-Cochran's test and t-Student's test for paired data were applied.

### RESULTS

The mechanical tests confirmed the impairment of wound healing in OJ, both in rats with 1-week extrahepatic cholestasis −2.50 *vs.* 1.66 ($p > 0,05$), as well as in 2-week obstructive jaundice −11.80 *vs.* 6.50 ($p > 0,001$). A lesser gain of body weight in jaundiced rats, in comparison to their controls, was also proven.

The results of histological evaluation of implanted polyurethane sponge discs in OJ and in control rats revealed a lack of early inflammatory reaction and a decrease of inflammatory cell number, but not statistically significant in the initial phase of healing in extracellular matrix, which was normalized or even showed tendency to increase in the implants of 2-week extrahepatic cholestasis. In contrast, the proliferation of fibroblasts (2.80 *vs.* 2.05), endothelial cells (2.80 *vs.* 1.95) and collagen deposition (2.80 *vs.* 1.60) were significantly lowered in 1-week OJ and even more so in rats with 2-week extrahepatic cholestasis in comparison to sham-operated controls (Fig. 1).

The biochemical studies with subcutaneously implanted polyurethane sponge disks in OJ rats revealed a decreased accumulation of hydroxyproline in 1-week (150.5 *vs.* 66.5) as well as in 2-week (414.5 *vs.* 164) implants of jaundiced rats in comparison to the sham-operated controls.

The results reflecting effects of OJ on the ability of peritoneal cells to produce TNF-$\alpha$ in culture are presented in Figure 2. The figure reveals several interesting features of peritoneal cells with regard to TNF-$\alpha$ production. Firstly,

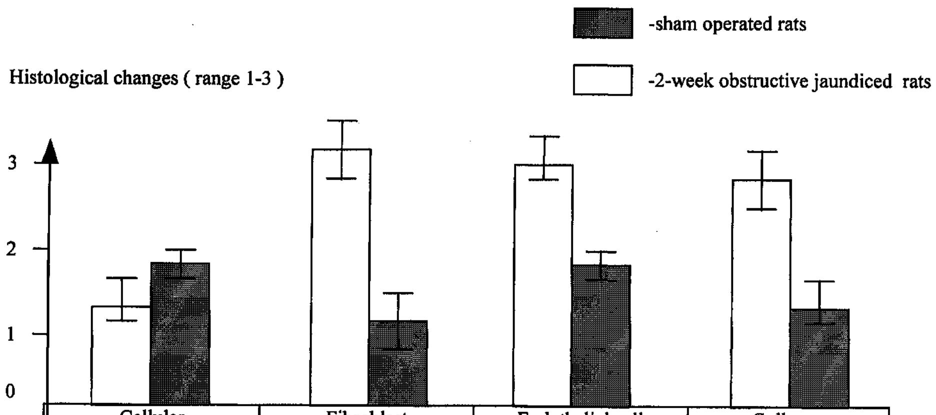

| | Cellular infiltration | Fibroblast proliferation | Endothelial cell proliferation | Collagen deposition |
|---|---|---|---|---|
| | 2-week obstructive jaundice | | | |
| Number of rats MV +/- SD | 10<br>1,75 +/- 0,54 | 23<br>1,25 +/- 0,49 | 20<br>1,65 +/- 0,47 | 23<br>1,25 +/- 0,48 |
| | Sham operated controls | | | |
| Number of rats MV +/- SD | 10<br>1,55 +/- 0,60 | 10<br>3,00 +/- 0,10 | 10<br>2,85 +/- 0,47 | 10<br>2,70 +/- 0,48 |
| Statistical differences | NSD | p.<0,01 | p.<0,001 | p.<0,001 |

FIGURE 1 Histological changes in polyurethane sponge implants of rats with 2-week obstructive jaundice.

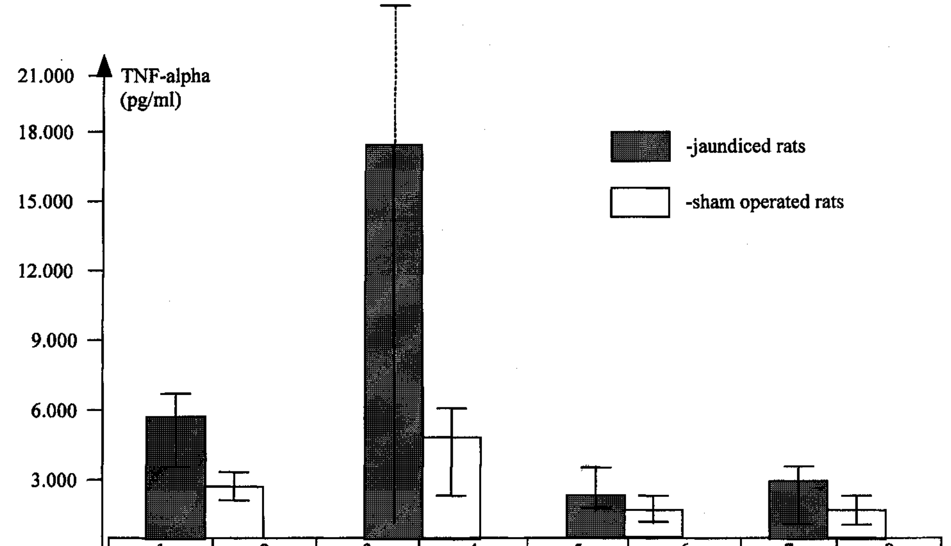

| | 1 | 2 | 3 | 4 | 5 | 6 | 7 | 8 |
|---|---|---|---|---|---|---|---|---|
| | 1-week obstructive jaundice | | | | 2-week obstructive jaundice | | | |
| Number of rats | 15 | 15 | 15 | 15 | 9 | 10 | 9 | 10 |
| MV +/- | 6,433 | 2,489 | 19,126 | 4,105 | 3,310 | 2,105 | 3,188 | 2,102 |
| SD | 4,494 | 1,946 | 19,513 | 2,691 | 1,8892 | 1,427 | 1,989 | 872 |
| Statistical differences | 1 : 5<br>3 : 7 | p.=0,06*<br>p.=0,02* | 2 : 6<br>4 : 8 | p.=0,60*<br>p.=0,03* | 1 : 2<br>3 : 4 | p.=0,004*<br>p.=0,006* | 5 : 6<br>7 : 8 | p.=0,13*<br>p.=0,13* |
| | 1 : 3 | p.=0,01** | 2 : 4 | p.=0,02** | 5 : 7 | NSD ** | 6 : 8 | NSD** |

FIGURE 2 TNF-alpha activity in peritoneal exudate cells of rats with obstructive jaundice and in sham operated controls.

the ability of cells of produce TNF-$\alpha$ on day 7 in OJ rats in much higher than on day 14. Secondly, the PEC from OJ rats on day 7, despite a high spontaneous TNF-$\alpha$ production, can be further, strongly stimulated by LPS for additional TNF-$\alpha$ production (a 3-fold increase). This is in contrast to day 14 of OJ where the spontaneous TNF-$\alpha$ production exceeds that of LPS-induced one. The cytokine production pattern in sham-operated rats resembles that in OJ rats *i.e.*, on day 7 PEC can be induced to a higher TNF-$\alpha$ production, although not to the same degree (less than 2-fold increase) and on day 14 the spontaneous TNF-$\alpha$ production is higher than LPS-inducible one. It should be also underlined that the induction of TNF-$\alpha$ production in both studied groups is highly statistically significant ($p = 0.01$ and 0.02, respectively), however, on day 14 these differences are not significant. In addition, the decreases of both spontaneous and LPS-induced TNF-$\alpha$ production between day 7 and 14 are significant.

### DISCUSSION

The process of wound healing in obstructive jaundice has been for a long time a subject of numerous studies [6, 17, 18, 24]. These studies were undertaken based on clinical observations indicating delay in wound healing and frequent local infections of wounds in patients with long-term OJ [6, 18]. Many experimental studies on animals with extrahepatic cholestasis confirmed these observations [16, 25]. Some authors, however, described opposite results [25].

Clinical evaluation of the healing process is difficult and the cause for obtaining different, often conflicting results, may derive from application of inadequate experimental models, not allowing objective, quantitative and qualitative assessment of the commonly accepted mechanical, histopathological and biochemical parameters of this process. Such criteria could not be fulfilled when preparations were taken from tissue sections derived from the marginal site of the wound. However, the model applied in our study, allowed to compare the studied parameters in a more objective manner. Our model of polyurethane sponge implants was used for the first time to investigate wound healing process in OJ.

The mechanical experiments on skin wound disruption strength confirmed the impairement of wound adhesion as well in 1-week, as in 2-week jaundiced rats and the observed differences were statistically significant. Our results are consistant with data of other authors [16, 17].

In the initial phase, the natural course of healing is associated with increased accumulation in the wound of various inflammatory cells, among them granulocytes, lymphocytes, plasmocytes, macrophages and endothelial cells. In our study in 7-day OJ the accumulation in the implants of inflammatory cells, compared with the control group, was distinctly decreased, although the differences were not statistically significant. Unequivocal explanation of this phenomenon is difficult, although it may be caused by disturbances in the early wound healing phase. Inhibition of inflammatory cell migration may be associated with activation of other alternative mediators of inflammation such as steroids or prostaglandins, fulfilling the role of protective, feedback mechanism factors in the response to endotoxaemia and increased TNF-alpha production. Such mechanisms may operate in surgical patients [25]. Other, *in vitro* studies, demonstrated a suppressive influence of endotoxin on TNF-alpha secretion by increased PGE-2 production, protecting the organism against damaging effects of cytokines [26].

Our studies confirmed suppressive effect of OJ on fibroblast and endothelial cell proliferation and accumulation of collagen deposition. Similarly, the content of hydroxyproline in the implants, a generally accepted indicator of the healing process and collagen production, was found lower both in 1-week as well as in 2-week OJ. The origin of these disturbances is not clear. In the early phase of wound healing, accompanied by extrahepatic cholestasis and systemic endotoxaemia, an increased reactivity of the immune system leads both to excessive TNF-alpha production as well as to activation of granulocytes and macrophages, resulting in release of free oxygen radicals and intracellular protease activation [14]. These reactions may cause impairment of the healing process leading to prolongation of the inflammatory phase in the wound and delay of the regeneration processess. The results, shown in this study, are in agreement with that finding. Perhaps the closest to our results are the data of Bemelmans *et al.* [27]. In an OJ mice model they demonstrated that the ability of macrophages from OJ mice to produce TNF-$\alpha$ spontaneously or upon LPS induction, as compared to sham-operated mice, was much higher as reported in our study. They also observed persisting, increased levels of TNF-$\alpha$ and IL-6 in serum of OJ mice which correlates with our unpublished data. Other studies, however [19, 20, 28] demonstrated that the ability of the immune system cell to produce cytokines and low molecular weight mediators is diminished. The reason for such a discrepancy remains unclear, it seems, however, that it results from the diversity of experimental models used.

Effect of obstructive jaundice on the process of wound healing constitutes only a fragment of all immunological abnormalities occuring in extrahepatic cholestasis associated with systemic endotoxemia, as a consequence of disturbance of cell immunity [7, 11, 19]. In the early stage of OJ an elevated reactivity of the immune system takes place resulting in an increased TNF-$\alpha$ production [8]. This was also confirmed in the present study on the spontaneous and LPS-induced cytokine production by peritoneal cells. The increased TNF-$\alpha$ synthesis, described in this report, could account for decreased collagen synthesis [29].

Studies *in vitro* or on local effects of TNF-$\alpha$ in various animal models revealed differential influence of this cytokine on selected parameters of the wound healing [15, 28, 30, 31]. According to Rapala *et al.*, who studied a local effect of TNF-$\alpha$ on the healing process, the cytokine was inhibitory as demonstrated by a decreased accumulation of inflammatory cells in granulation tissue, confined only to the initial phase of the healing process [15]. Mooney *et al.*, in normal and adriamycin-impaired mice described an opposite phenomenon and demonstrated a beneficial influence of a local application of TNF-$\alpha$ in the healing process, as determined by wound disruption strength. However, in this type of experiments, collagen was used as a carrier for TNF-$\alpha$ and that phenomenon was dose-dependent [31]. Unfortunately, such type of experiments, where egzogenous TNF-$\alpha$ is locally applied, cannot fully explain the role of TNF-$\alpha$ in wound healing. It seems rather, that demonstration of the ability of RES cells to produce this cytokine is more relevant.

Two phenomena associated with TNF-$\alpha$ production by PEC, presented in this work, require special attention since they have been not reported before in analysis of the cytokine response profile in OJ animals. The first one is the inducibility of TNF-$\alpha$ production on day 7 and lack of it on day 14. Second, it is evident that the operation itself causes different responsiveness of PEC on day 7 and 14. The first phenomenon may be caused by a desensitization of RES cells (day 14) following prolonged endotoxin stimulation, preceded by a peak of stimulation of the cells on day 7. The effect of operation alone seems to be also evident resulting in the unresponsiveness of cells from sham operated rats to LPS stimulation (day 14). This is probably caused by surgery-elicited cytokine production [32] which we have described for splenectomized mice. The latter phenomenon has probably some influence on the cytokine pattern in OJ rats where we face an additive effect of surgery and endotoxin stimulation, so that the resultant picture may be obscured. It seems that the involvement of TNF-alpha during the process of wound healing is differential and depends on the phase of this process. In the early stages TNF-$\alpha$ stimulates fibroblast proliferation and collagen deposition, but in the later phase of wound healing this cytokine is inhibitory with regard to collagen synthesis [31]. Such behaviour of TNF-alpha may just reflect the regulatory nature of this cytokine. There is, however, no doubt that TNF-alpha is essential for fibroblast proliferation [33, 34], angiogenesis [35] and generally for defence processes [9] and wound healing [32].

In conclusion, the results of this report on mechanical, histopathological and biochemical parameters of the healing process in OJ indicate its impairment and the important role for extrahepatic cholestasis and systemic endotoxaemia. Immunological studies showed that disturbances in functioning of the immune system may be the main cause of the delay in the healing process of wounds in obstructive jaundice.

### *References*

[1] Fogarty, B. J., Parks, R. W., Rowlands, B. J. and Diamond, T. (1995). Renal dysfunction in obstructive jaundice. *British Journal of Surgery*, **82**, 877–884.

[2] Pain, J. A., Cahill, C. J. and Bailey, M. E. (1985). Perioperative complications in obstructive jaundice: therapeutic considerations. *British Journal of Surgery*, **72**, 535–538.

[3] Dawiskiba, J. (1996). The role of endotoxaemia in the development of renal disorders in experimental obstructive jaundice in rats. *Journal of HPB Surgery*, **10**, 7–10.

[4] Sagar, S. and Shields, R. (1980). Fibrinogen in the hepato-renal syndrome: an experimental study. *British Journal of Surgery*, **67**, 562–564.

[5] Greig, J. D., Krukowski, Z. H. and Matheson, N. A. (1988). Surgical morbidity and mortality in one hundred and twenty nine patients with obstructive jaundice. *British Journal of Surgery*, **75**, 216–219.

[6] Grande, L., Garcia-Valdecasas, J. C., Fuster, J., Visa, J. and Pera, C. (1990). Obstructive jaundice and wound healing. *British Journal of Surgery*, **77**, 440–442.

[7] O'Connor, M. J. (1985). Mechanical biliary obstruction. A review of the multisystemic consequences of obstructive jaundice and their impact on perioperative morbidity and mortality. *The American Surgeon*, **51**, 245–251.

[8] Greve, J. W., Gouma, D. J., Soeters, P. B. and Buurman, W. A. (1990). Suppression of cellular immunity in obstructive jaundice is caused by endotoxins: a study with germ-free rats. *Gastroenterology*, **98**, 478–485.

[9] Thompson, R. L. E., Hoper, M., Diamond, T. and Rowlands, B. J. (1990). Development and reversibility of T lymphocyte dysfunction in experimental obstructive jaundice. *British Journal of Surgery*, **77**, 1229–1232.

[10] Saitoh, N., Hiraoka, T., Uchino, R. and Miyauchi, Y. (1995). Endotoxemia and intestinal mucosal dysfunction after the relief of obstructive jaundice by internal and external drainage in rats. *European Surgical Research*, **27**, 11–18.

[11] Clements, W. D. B., Halliday, I., McCaigue, M. D., Barcley, R. G. and Rowlands, B. J. (1993). Effects of extrahepatic obstructive jaundice on Kupffer cell clearance capacity. *Archives of Surgery*, **128**, 200–205.

[12] Hori, Y. and Ohyanagi, H. (1997). Protective effect of the intravenous administration of ursodeoxycholic acid against endotoxemia in rats with obstructive jaundice. Surgery Today. *Japan Journal Surgery*, **27**, 140–144.

[13] Van Bossuyt, H., Desmaretz, C., Gaeta, G. B. and Wisse, E. (1990). The role of bile acids in the development of endotoxemia during obstructive jaundice in the rat. *Journal of Hepatology*, **10**, 274–279.

[14] Ding, A. H., Nathan, C. F. and Stuehr, D. J. (1988). Release of reactive nitrogen intermediates and reactive oxygen intermediates from the mouse peritoneal macrophages. Comparison of the activating cytokines and evidence for independent production. *Journal of Immunology*, **141**, 2407–2412.

[15] Rapala, K., Laato, M., Niikikoski, J., Kujari, H., Soder, O., Mauviel, A. and Pujol, J. P. (1991). Tumor necrosis factor alfa inhibits wound healing in the rat. *European Surgical Research*, **23**, 261–268.

[16] Askew, A. R., Bates, G. J. and Balderson, G. (1984). Jaundice and the effect of sodium taurocholate taken upon abdominal wound healing. *Surgery, Gynecology and Obstetrics*, **159**, 207–209.

[17] Arnaud, J. P., Humbert, W., Eloy, M. R. and Adloff, M. (1981). Effect of obstructive jaundice on wound healing in experimental study in rats. *American Journal of Surgery*, **141**, 593–596.

[18] Amstrong, C. P., Dixon, J. M., Duffy, S. W., Elton, R. A. and Davies, G. C. (1984). Wound healing in obstructive jaundice. *British Journal of Surgery*, **71**, 267–270.

[19] Houdijk, A. P. J., Boermeester, R. I. C., Wesdorp, C., Hack, C. E. and Van Leeuwen, P. A. M. (1996). Tumor necrosis factor unresponsiveness after surgery in bile duct ligated rats. *American Journal Physiology*, **271**, 980–986.

[20] Reynolds, J. V., Murchan, P., Redmond, H. P., Watson, R. W. G., Leonard, P., Hill, A., Clarke, P., Marks, P., Keane, F. B. V. and Tanner, W. A. (1995). Failure of macrophage activation in experimental obstructive jaundice: association with bacterial translocation. *British Journal of Surgery*, **85**, 534–538.

[21] Woessner, J. F. (1961). The determination of hydroxyproline in tissue and protein samples containing small proportions of this aminoacid. *Archives of Biochemistry and Biophysics*, **93**, 440–447.

[22] Espevik, T. and Nissen-Meyer, J. (1986). A highly sensitive cell line, WEHI 164 clone for measuring cytotoxic factor/tumor necrosis factor from human monocytes. *Journal of Immunological Methods*, **95**, 99–105.

[23] Hansen, M. B., Nielsen, S. E. and Berg, K. (1989). Reexamination and further development of a precise and rapid dye method for measuring cell growth/kill. *J. Immunol. Meth.*, **119**, 203–210.

[24] Greaney, M. G., Van Noort, R., Smythe, A. and Irvin, T. T. (1979). Does Obstructive jaundice adversely affect wound healing? *British Journal of Surgery*, **66**, 478–481.

[25] Naito, Y. (1992). Responses of plasma Adrenocortycotropic hormone, Cortisol and cytokines during and after upper abdominal surgery. *Anaesthesiology*, **77**, 426–431.

[26] Callery, M. P., Mangino, M. J. and Flye, M. W. (1991). Arginine-specific suppresion of the mixed lymphocyte culture reactivity by Kupffer cells–a basis of portal venous tolerance. *Transplantation*, **51**, 1076–1080.

[27] Bemelmans, M. H. A., Gouma, D. J., Greve, J. W. and Buurman, W. A. (1992). Cytokines tumor necrosis factor and interleukin 6 in experimental biliary obstruction in mice. *Hepatology*, **15**, 1132–1138.
[28] Steenfos, H. H., Hunt, T. K., Schenenstuhl, M. and Goodson, W. H. (1989). Selective effects tumor necrosis factor-alfa on wound healing in rats. *Surgery*, **106**, 171–176.
[29] Regan, M. C., Kirk, S. J., Hursen, M., Sodeyama, M., Wasserkrug, H. L. and Barbul, A. (1993). Tumor necrosis factor alpha inhibits in vivo collagen synthesis. *Surgery*, **113**, 173–177.
[30] Bettinger, D. A., Pellicane, J. V., Tarry, W. C., Yager, D. R., Diegelmann, R. F., Lee, R., Cohen, I. K. and Demaria, E. J. (1994). The role of inflammatory cytokines in wound healing: accelerated healing in endotoxin-resistant mice. *Journal of Trauma*, **36**, 810–813.
[31] Moonery, D. P., O'Reilly, M. and Gamelli, R. L. (1990). Tumor necrosis factor and wound healing. *Annals of Surgery*, **211**, 124–129.
[32] Zimecki, M., Właszczyk, A., Zagulski, T. and Kübler, A. (1998). Lactoferrin lowers interleukin 6 and tumor necrosis factor alpha serum levels in mice subjected to surgery. *Archivum Immunologiae et Therapiae Experimentalis*, **46**, 97–104.
[33] Solis-Herruzo, J., Brenner, D. A. and Chojkier, M. (1988). Tumor necrosis factor alpha inhibits collagen gene transcription and collagen synthesis in cultured human fibroblasts. *Journal of Biological Chemistry*, **263**, 5841–5845.
[34] Vilcek, J., Palombella, V., Henriksen-De Stefano, D. *et al.* (1986). Fibroblast growth enhancing activity of tumor necrosis factor and its relationship to other polypeptide growth factors. *Journal of Experimental Medicine*, **163**, 632–643.
[35] Leibovich, S. J., Polverini, J. P., Shepard, H. M., Wiseman, D. M., Shively, V. and Nurredin, N. (1987). Macrophage-induced angiogenesis is mediated by tumor necrosis factor-alpha. *Nature*, **329**, 630–632.

### SUMMARY

In rats in 1-week and 2-week obstructive jaundice and in their sham operated controls the wound healing process and activity of TNF-alpha in PEC were investigated. In the study the skin wound breaking strength was evaluated. Also histologically and biochemically polyurethane sponge disks implanted subcutaneously into the rats were studied. In implants the hydroxyproline content and inflammative cellular infiltration, fibroblast and endothelial cell proliferation and collagen deposition were estimated. Additionly in PEC culture the activity of TNF-alpha was measured.

In rats with obstructive jaundice the impairment the both early and late phase of healing process were impaired. The breaking strength of skin wound were decreased, the fibroblast and endothelial cell proliferation and collagen deposition were depressed, as well as hydroxyproline content. In 1-week obstructive jaundice the number of inflammatory cells in implants was diminished, which slighty increased in 2-week extrahepatic cholestasis. The spontaneous and endotoxinstimulated activity of TNF-alpha in PEC was significantly higher in rats with 1-week obstructive jaundice, but its level decreased in 2-week extrahepatic cholestasis.

The impairment of wound healing in the early phase of obstructive jaundice is explained by hyperreactivity of the immunological system and increased production of proinflammatory cytokines followed by hyporeactivity to endotoxin in later phase of jaundice. The main cause of this disorder is systemic endotoxaemia in obstructive jaundice.

